# Short ‘1.2× Genome’ Infectious Clone Initiates Kolmiovirid Replication in *Boa constrictor* Cells

**DOI:** 10.3390/v14010107

**Published:** 2022-01-08

**Authors:** Leonora Szirovicza, Udo Hetzel, Anja Kipar, Jussi Hepojoki

**Affiliations:** 1 Medicum, Department of Virology, University of Helsinki, 00290 Helsinki, Finland; jussi.hepojoki@helsinki.fi; 2 Institute of Veterinary Pathology, Vetsuisse Faculty, University of Zürich, 8057 Zürich, Switzerland; udo.hetzel@uzh.ch (U.H.); anja.kipar@uzh.ch (A.K.); 3 Department of Veterinary Biosciences, Faculty of Veterinary Medicine, University of Helsinki, 00790 Helsinki, Finland

**Keywords:** deltavirus, infectious clone, HDV

## Abstract

Human hepatitis D virus (HDV) depends on hepatitis B virus co-infection and its glycoproteins for infectious particle formation. HDV was the sole known deltavirus for decades and believed to be a human-only pathogen. However, since 2018, several groups reported finding HDV-like agents from various hosts but without co-infecting hepadnaviruses. In vitro systems enabling helper virus-independent replication are key for studying the newly discovered deltaviruses. Others and we have successfully used constructs containing multimers of the deltavirus genome for the replication of various deltaviruses via transfection in cell culture. Here, we report the establishment of deltavirus infectious clones with 1.2× genome inserts bearing two copies of the genomic and antigenomic ribozymes. We used Swiss snake colony virus 1 as the model to compare the ability of the previously reported “2× genome” and the “1.2× genome” infectious clones to initiate replication in cell culture. Using immunofluorescence, qRT-PCR, immuno- and northern blotting, we found the 2× and 1.2× genome clones to similarly initiate deltavirus replication in vitro and both induced a persistent infection of snake cells. The 1.2× genome constructs enable easier introduction of modifications required for studying deltavirus replication and cellular interactions.

## 1. Introduction

Hepatitis D virus (HDV) is a unique human pathogen. Three years after its discovery in 1977 in liver specimens of chronically hepatitis B (HBV)-infected patients [1], Rizzetto and colleagues identified it as a satellite virus of HBV [2]. One can contract HDV in two different ways, either through acute co-infection with HBV or through superinfection as a chronic HBV carrier. HBV and HDV co-infection is clinically more severe than HBV mono-infection; however, the infection usually resolves, resulting in the clearance of both viruses. Superinfection of a chronic HBV carrier by HDV results in the most severe form of viral hepatitis; these patients often face hepatic cirrhosis and development of hepatocellular carcinoma [3]. HDV is a satellite virus that utilizes the envelope proteins of HBV to assemble infectious viral particles; however, the replication of HDV within the host cell proceeds independently of HBV [4]. The single-stranded RNA genome of HDV is around 1.7 kilonucleotides (knt) long, although because of high self-complementary, it forms a double-stranded rod-like structure [5,6]. Within the cell, HDV gives rise to three different RNA species: the genome, the antigenome, and the mRNA. The antigenome is the exact complement of the genome, while the mRNA mediates the expression of the delta antigen (DAg), the sole protein encoded by the HDV genome [5,7]. During the viral life cycle, the DAg is present in two different forms, small (SDAg) and large DAg (LDAg) [8]. Cellular editing mediated by adenosine deaminase acting on RNA (ADAR1) converts the amber stop codon of the SDAg on the antigenomic strand to a tryptophan codon, thus allowing the extension of the protein by 19 additional amino acids [9,10]. The two forms of the protein not only differ in their length but they also have vastly different roles. The SDAg promotes viral replication, while the LDAg inhibits it and shifts the viral life cycle towards packaging [8]. The host cell’s RNA polymerases mediate the replication of the HDV genome, which occurs via a double rolling circle mechanism [11]. A curious feature of HDV is the presence of ribozyme sequences in both its genome and antigenome [12]. The ribozymes cut the multimeric HDV RNA species produced during the rolling circle replication into unit-length pieces [11].

HDV was the sole representative of the unassigned genus *Deltavirus* until 2018 [13]. The discovery of HDV-like sequences in birds and snakes in 2018 marked the beginning of a new chapter in deltavirus research by broadening the potential host spectrum [14,15]. First, an HDV-like sequence was discovered in waterfowl during a meta-transcriptomic study without traces of HBV or hepadnaviral reads but influenza A virus reads instead [14]. Co-incidentally, we reported the identification of a deltavirus in *Boa constrictors* (Swiss snake colony virus 1, SwSCV-1, initially known as snake deltavirus, SDeV) in co-infection with reptarena- and hartmaniviruses but in the absence of hepadnaviral reads [15]. The next paradigm shift of deltavirus research was the broadening of the scope of putative HDV helper viruses to include vesiculo-, flavi-, and hepaciviruses [16]. The researchers further showed that HDV forms infectious particles using the glycoproteins (GPs) of the aforementioned viruses, not only in liver, but also in kidney cells. Soon after, we managed to isolate SwSCV-1 along with co-infecting reptarena- and hartmaniviruses from brain homogenates of an infected snake [17]. We utilized persistently SwSCV-1-infected cell cultures to demonstrate that reptarena- or hartmanivirus superinfection results in the egress of infectious SwSCV-1 particles, adding to the evidence that reptarena- and hartmaniviruses are the likely helpers of the virus [17]. We further demonstrated that expression of arena- and orthohantavirus GPs in the persistently infected cultures also induces infectious particle formation [17]. The aforementioned reports provoked several metatranscriptomic studies, resulting initially in the identification of deltaviruses in subterranean termites, fish, Asiatic toad, and Chinese fire belly newt in 2019 [18]. In 2020, Paraskevopoulou and colleagues found a deltavirus in Tome’s spiny rats without sequences for a helper virus, but including evidence for autonomous replication in cell culture and the host [19]. Metatranscriptomic studies revealed more deltaviruses in common vampire bats, a lesser dog-like bat, white-tailed deer, eastern woodchuck, passerine birds, zebra finch, lantern fish, and amboli leaping frogs [20,21,22]. The recent findings have sparked deltavirus research and raised questions about the evolutionary origin of deltaviruses [23,24,25]. The identification of deltaviruses across several taxa led to the establishment of a new realm, *Ribozyviria*, with family *Kolmioviridae* including eight genera [26,27].

A number of tools to initiate HDV replication in vitro and to mimic infection have been developed over the years. The first and probably most widely used method is the transfection of cells with a plasmid containing a trimer of the entire HDV genome under the control of the simian virus 40 promoter [4]. In theory, the transfection results in multimeric RNA transcripts of the HDV genome, which resembles the rolling circle replication of the virus during actual infection. It is also possible to initiate the replication by transfecting a vector carrying a monomer of the HDV genome, but in this case, an HDAg-expressing plasmid needs to be provided *in trans* [28]. Macnaughton and Lai showed that direct RNA transfection with HDAg provided *in trans* can overcome the use of artificial DNA intermediates to initiate replication. Interestingly, they found that transfection with 1.2× genome-length RNA resulted in the most efficient replication, perhaps because the ribozymes at both ends of the genome were in duplicate [29]. In our previous study, we constructed a plasmid containing the entire SwSCV-1 genome in duplicate in head-to-tail fashion [17], following the idea of the trimeric HDV constructs [4]. The transfection of the “2×” construct initiates efficient SwSCV-1 replication in snake cells, eventually leading to persistent infection [17]. The “genome-dimer or 2×” plasmid principle was also successfully adapted for Tome’s spiny rat virus 1 (TSRV-1), *Taeniopygia guttata* deltavirus, and *Marmota monax* deltavirus [19,21]. Here, we describe a construct containing 1.2× SwSCV-1 genome, which, in a manner similar to the previously described 2× SwSCV-1 infectious clone [17], initiates virus replication, produces infectious particles upon superinfection with Haartman institute snake virus 1 (HISV-1), and results in persistent infection of the cells. Because we did not manage to decipher the exact compositions of the plasmid-based 1.2× [30] and 1.1× [31] genome constructs reported earlier, we based our 1.2× infectious clone on the RNA transfection studies of Macnaughton and Lai [29] with the aim to generate shorter DNA constructs to facilitate introduction of mutations or modifications to the virus genome. Additionally, we wanted to eliminate the T7 promoter we used in the 2× SwSCV-1 infectious clone to avoid the risk of DAg expression via this promoter due to cellular polymerases, which is reported to occur in mammalian cells [32,33].

## 2. Materials and Methods

### 2.1. Cell Culture and Superinfection

The study made use of previously described cultured *Boa constrictor* kidney cells, I/1Ki, [34] and persistently SwSCV-1- infected I/1Ki cells, I/1Ki-Δ [17]. The cells were maintained in Minimal Essential Medium Eagle (Sigma-Aldrich, St.Louis, MO, USA) supplemented with 10% fetal bovine serum (ThermoFisher Scientific, Waltham, MA, USA), 200 mM L-glutamine (Sigma-Aldrich, St. Louis, MO, USA), 100 µg/mL of streptomycin (Sigma-Aldrich, St. Louis, MO, USA), and 100 U/mL of penicillin (Sigma-Aldrich, St. Louis, MO, USA) in an incubator at 30 °C with 5% CO_2_.

To establish another persistently SwSCV-1-infected I/1Ki cell line, designated I/1Ki-1.2×Δ, we transfected I/1Ki cells with a plasmid containing 1.2 copies of the SwSCV-1 genome (described below) and maintained the cells as described above and earlier [17].

To study infectious particle formation of the SwSCV-1-infected cell lines, we conducted superinfection studies with HISV-1 [35], earlier demonstrated to be an efficient helper virus for SwSCV-1 [17]. The superinfection studies and detection followed the protocol described [17]. For the infection, we used 600 copies of HISV-1 S segment RNA per cell, which corresponds roughly to a multiplicity of infection (MOI) of 10.

### 2.2. Plasmids and Cloning

**Figure 1:**
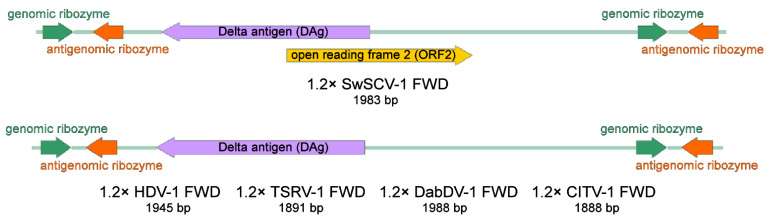
Schematic representation of the “1.2× genome” kolmiovirid inserts in forward (FWD)/genomic orientation. We cloned the 1.2× genome of the displayed kolmiovirid: Swiss snake colony virus 1 (SwSCV-1, GenBank accession: NC_040729.1, 1.15× genome), human hepatitis D virus genotype 1 (HDV-1, M21012.1, 1.16× genome), Tome’s spiny rat virus 1 (TSRV-1, MK598005.2, 1.13× genome), Dabbling duck virus 1 (DabDV-1, NC_040845.1, 1.17× genome), and Chusan Island toad virus 1 (CITV-1, MK962760.1, 1.22× genome) into pCAGGS/MCS plasmid, both in genomic (FWD—shown in this figure) and in antigenomic (REV) orientation. Each of the inserts, approximately 1.2× of the genome size, contains a single copy of the genomes flanked from each end by both the genomic and antigenomic ribozymes. The images were created using SnapGene Viewer (https://www.snapgene.com/snapgene-viewer/; accessed on 14 October 2019).

We ordered synthetic genes from Gene Universal for the 1.2× copies of the following kolmiovirids: SwSCV-1 (initially known as snake deltavirus, GenBank accession number: NC_040729.1) [17], Tome’s spiny rat virus 1 (TSRV-1, initially known as rodent deltavirus, MK598005.2) [19], dabbling duck virus 1 (DabDV-1, initially known as avian HDV-like agent, NC_040845.1) [14], Chusan Island toad virus 1 (CITV-1, initially known as toad HDV-like agent, MK962760.1) [18], and HDV-1 (M21012.1). Each synthetic gene, flanked by EcoRV restriction sites, contained the full genome with additional nucleotides to include both the genomic and antigenomic ribozymes twice (Figure 1). Subcloning of the constructs into pCAGGS followed the procedures described in Szirovicza et al., 2020 [17]. Briefly, FastDigest EcoRV (ThermoFisher Scientific, Waltham, MA, USA) served to restriction digest the inserts, followed by purification after agarose gel electrophoresis using the GeneJET Gel extraction kit (ThermoFisher Scientific, Waltham, MA, USA). T4 DNA ligase (ThermoFisher Scientific, Waltham, MA, USA) served to ligate the purified inserts into pCAGGS/MCS plasmid [36] purified from agarose gel by the GeneJET Gel extraction kit (ThermoFisher Scientific, Waltham, MA, USA) after linearization with FastDigest EcoRI and XhoI (ThermoFisher Scientific, Waltham, MA, USA) restriction enzymes and T4 DNA polymerase (ThermoFisher Scientific, Waltham, MA, USA) blunting. We plated chemically competent *Escherichia coli* (DH5α strain) transformed with the ligation products on Luria Broth (LB) agar plates with 100 µg/mL of ampicillin, and incubated overnight (O/N) at 37 °C. We picked single colonies and transferred them into 5 mL of LB medium (10 g/L tryptone, 10 g/L NaCl, 5 g/L yeast extract), followed by O/N incubation at 37 °C (220 rpm), after which the GeneJET Plasmid Miniprep Kit (ThermoFisher Scientific, Waltham, MA, USA) served for plasmid isolation from 2 mL of the O/N culture. The DNA Sequencing and Genomic Laboratory, Institute of Biotechnology, University of Helsinki performed Sanger sequencing of the preparations and confirmed these to contain an insert of the correct size. For each virus, we selected two clones, one each with the insert in genomic and in antigenomic orientation, for plasmid stock preparation using ZymoPURE II Plasmid Maxiprep Kit (Zymo Research, Irvine, CA, USA).

### 2.3. Transfection

Lipofectamine 2000 (ThermoFisher Scientific, Waltham, MA, USA) reagent served for transfection of I/1Ki cells as described [17,37]. Briefly, we mixed 500 ng of plasmid DNA in 50 µL of OptiMEM (ThermoFisher Scientific, Waltham, MA, USA) and 3 µL of Lipofectamine 2000 in 47 µL of OptiMEM (ThermoFisher Scientific, Waltham, MA, USA) by pipetting up and down, and allowed the complexes to form for 15–30 min at room temperature (RT). We added 1 mL of trypsinized cells (suspension containing approximately 1.8 cm^2^ of cells per ml) to the mixture and allowed the suspension to stand at RT for 15–30 min before plating. At 5–6 h post plating, we replaced the transfection mixture by fully supplemented medium and incubated the cells as described above. We scaled up the above reaction volumes depending on the amount of cells needed for each experiment.

### 2.4. Western Blot (WB)

For WB, we washed the cells grown on plates or flasks twice with PBS, scraped them into PBS, pelleted by centrifugation (500× *g*, 3–5 min), lysed the cell pellets by RIPA buffer (50 mM Tris, 150 mM NaCl, 1% Tx-100, 0.1% SDS, 0.5% sodium deoxycholate, protease inhibitor cocktail), and measured the protein concentration using the Pierce^TM^ BCA Protein Assay Kit (ThermoFisher Scientific, Waltham, MA, USA). For comparing DAgs of different kolmiovirids, we collected from a 12-well plate nontransfected I/1Ki cells and those transfected with FWD and REV constructs at 4 days post transfection in 100 µL of Laemmli sample buffer after two washes with PBS. We separated an equal amount of protein (or volume, 30 µL/lane, for the experiments done on 12-well plate) for each sample on SDS-PAGE using 4–20% Mini-PROTEAN^®^ TGX gels (Bio-Rad, Hercules, CA, USA), and immunoblotted as described [38]. We used 1:4000 dilution for the rabbit anti-SDAg primary antibody [15] or 1 µg/mL of IgG affinity purified from anti-SwSCV-1 rabbit antiserum [17] using recombinant human DAg as described [17], 1:10,000 for AlexaFluor 680 donkey anti-mouse (IgG) (ThermoFisher Scientific, Waltham, MA, USA), and 1:10,000 for IRDye 800CW donkey anti-rabbit (IgG) (LI-COR Biosciences, Lincoln, NE, USA). The Lab Vision^TM^ pan-actin mouse monoclonal antibody (ThermoFisher Scientific, Waltham, MA, USA) used at a 1:200 dilution served for detection of β-actin. The Odyssey Infrared Imaging System (LI-COR Biosciences, Lincoln, NE, USA) was employed to record the results.

### 2.5. Immunofluorescence Staining

For immunofluorescence (IF) staining, we plated the cells on collagen coated (10 µg/cm^2^ type I rat tail collagen (BD Biosciences, Franklin Lakes, NJ, USA) in 25 mM acetic acid, O/N at 4 °C) CellCarrier-96 Ultra plates (PerkinElmer, Waltham, MA, USA). After the removal of culture media, cells were fixed by incubation in 4% paraformaldehyde in PBS for ~15 min at RT. The IF staining followed the protocol described [17]. We used directly labeled anti-SDAg-AF488 [17] at 1:500 dilution, rabbit anti-SDAg antiserum at 1:4000 dilution for detection of SwSCV-1 and 1:100 dilution for the detection of HDV, TSRV-1, DabDV-1, CITV-1, and for the clean/nontransfected I/1Ki cells, and 1:1000 of either Alexa Fluor 488- or 594-labeled donkey anti-rabbit immunoglobulin (ThermoFisher Scientific, Waltham, MA, USA) as the secondary antibody. The Opera Phenix High Content Screening System (PerkinElmer, Waltham, MA, USA), provided by FIMM (Institute for Molecular Medicine Finland) High Content Imaging and Analysis (FIMM-HCA, Helsinki, Finland), served for imaging of the plates stored in the dark at 4 °C.

### 2.6. Detection of Circular RNA Genome

To show the circularity of the SwSCV-1 genome, we performed reverse transcription (RT) with two different SwSCV-1-specific primers: RT-1 5′-GTTTCCCCACAAATTCTTTGC-3′; RT-2 5′-CCTCTATCCTACTTCAATTCTC-3′. For the cDNA synthesis, we used SuperScript™ IV Reverse Transcriptase (ThermoFisher Scientific, Waltham, MA, USA) according to the manufacturer’s recommendations. The cycling conditions for the RT reaction were the following: 5 min at 50 °C, 15 min at 55 °C, 10 min at 60 °C, and 15 min at 65 °C. Subsequently, we used three different primer pairs (PP) with neighboring 5′ ends, but their 3′ ends facing opposite directions, similarly to the method used by Paraskevopoulou et al. for TSRV-1 [19]. With such PPs, one should only be able to amplify a product if the RNA template is circular. The PPs were the following: PP1 forward 5′-GGATCTGCTTCTTGGATGGAGTTTCC-3′, PP1 reverse 5′- GAAGAAGAGAAAGCTTGAGGAGCAGC-3′; PP2 forward 5′- GCTTCTGCTCCTTGCCTCTCAC-3′, PP2 reverse 5′- GGCTCGAGGCTACCACCGAAAGAG-3′; PP3 forward 5′- GGTTCACTTCCCCAGCTCCTC-3′, PP3 reverse 5′- CGGGACTAGACGTGAGGGGTG-3′. To amplify the genome starting from the DAg ORF, we used Phusion Flash High-Fidelity PCR Master Mix (ThermoFisher Scientific, Waltham, MA, USA) according to the manufacturer’s protocol with the following cycling conditions: initial denaturation for 1 min at 98 °C, three-step amplification at 98 °C for 1 s, 68 °C for 5 s, 72 °C for 35 s repeated for 35 cycles, and the final elongation for 30 s at 72 °C.

### 2.7. Quantitative Reverse Transcription PCR (qRT-PCR)

qRT-PCR served for quantification of viral RNA in the cells. The primers and probe were the following: forward primer 5′-GAAAGACGCGACAACTGTGAGTC-3′, reverse primer 5′-GTCTAGTCCCGTTCCGGTTCTATG-3′, and probe 5′ 6-Fam (carboxyfluorescein)-GGAGATCCGAGAGGGGAGAAGAGGAGAGGTC-BHQ (black hole quencher)-1 3′, which target SwSCV-1 RNA in genomic orientation. We isolated RNA for qRT-PCR using the GeneJET RNA Purification Kit (ThermoFisher Scientific, Waltham, MA, USA) with the addition of carrier RNA when purifying RNA from cell culture supernatants. We used TaqMan^®^ Fast Virus 1-Step Master Mix (ThermoFisher Scientific, Waltham, MA, USA) to set up 10 µL (half volume) reactions according to the manufacturer’s recommendations with the addition of 8% DMSO to prevent secondary structure formation. The AriaMX real-time PCR system (Agilent, Santa Clara, CA, USA) served for thermal cycling of the duplicate samples with the recommended conditions: reverse transcription for 5 min at 50 °C; initial denaturation for 20 s at 95 °C; two amplification steps at 95 °C for 3 s and 60 °C for 30 s repeated for 40 cycles.

To generate a control RNA for copy-level quantification, we used the SwSCV-1 FWD plasmid described in Szirovicza et al., 2020 [17]. Briefly, FastDigest SmaI (ThermoFisher Scientific, Waltham, MA, USA) following manufacturer’s protocol served for linearization of the plasmid. The GeneJet Gel Purification Kit (ThermoFisher Scientific, Waltham, MA, USA) served for purification of the linearized plasmid after agarose gel separation. We used the TranscriptAid T7 High Yield Transcription Kit (ThermoFisher Scientific, Waltham, MA, USA) according to the manufacturer’s protocol to in vitro transcribe the target RNA. Subsequently, we purified the RNA by the GeneJET RNA Purification Kit (ThermoFisher Scientific, Waltham, MA, USA), diluted it into diethyl pyrocarbonate-treated water, and stored the RNA in aliquots at −80 °C until use. The NanoDrop 2000 spectrophotometer (ThermoFisher Scientific, Waltham, MA, USA) served for quantification of the control RNA, and an online copy number calculator (http://endmemo.com/bio/dnacopynum.php; accessed on 16 December 2021) for converting the concentration to RNA copies per microliter. We ran the control RNA as 10-fold dilution series in duplicates for each run to generate a standard curve for estimation of RNA copy numbers in cell and cell culture supernatant samples. 

To normalize the SwSCV-1 RNA levels against a house-keeping gene, we ordered the following primers and probe for the detection of *Boa constrictor* glyceraldehyde-3-phosphate dehydrogenase (GAPDH): forward primer: 5′ CTGGTATGACAACGAATA 3′, reverse primer: 5′ CAGTCTTTACTCCTTAGATG 3′, and probe 5′ 6-Fam (carboxyfluorescein)-TGAACCAACAAGTCTACCACACG-BHQ-1 3′. Reference assembly using *Python bivitattus* GAPDH (GenBank accession: XM_007429612.3) as the template in Unipro UGENE (http://ugene.net/; accessed on 11 July 2019) [39] served to obtain the mRNA for *B. constrictor* GAPDH from the reads of our earlier metatranscriptomic studies [35,40,41,42]. 

### 2.8. Near-Infrared Fluorescent Northern Blot 

For northern blot analysis, TRIzol^TM^ reagent (ThermoFisher Scientific, Waltham, MA, USA) used according to the manufacturer’s recommendations served for isolating RNA from either T75 or T175 flasks of I/1Ki, I/1Ki-2×Δ, and I/1Ki-1.2×Δ. The samples were solubilized either into formamide or into milliQ water. The NanoDrop 2000 spectrophotometer (ThermoFisher Scientific, Waltham, MA, USA) was employed for quantification of the RNA. Subsequently, we ran 3–5 µg of RNA to detect genomic SwSCV-1 RNA, 15–20 µg to detect antigenomic RNA, and an ssRNA ladder (New England Biolabs, Ipswich, MA, USA) on agarose–formaldehyde gels using the tricine/triethanolamine buffer system described by Mansour and Pestov [43]. We employed two different loading dyes to prepare the RNA samples for the run, either the one described by Mansour and Pestov [43] and prepared “in-house” or the 2X RNA Loading Dye provided with the ssRNA ladder (New England Biolabs). Then, we transferred the RNAs from the gel to Hybond^TM^-N^+^ nylon membrane (GE Healthcare, Chicago, IL, USA) by capillary transfer O/N and cross-linked the RNA to the membrane by an ultraviolet cross-linker (120 mJ/cm^2^ at 254 nm) (Analytik Jena, Jena, Germany). After RNA cross-linking, we stained some of the membranes with 0.02% methylene blue in 0.3M sodium acetate, pH 5.5, to visualize the bands. Incubation in prehybridization buffer (5× sodium saline citrate buffer (SSC), 5× Denhardt’s solution (ThermoFisher Scientific, Waltham, MA, USA), 1% SDS, 50% formamide and 100 µg ultrapure herring sperm DNA (ThermoFisher Scientific, Waltham, MA, USA)) at 68 °C or 48 °C (depending on the probe) for 2–4 h served for blocking the nonspecific binding sites on the membrane. The following probes (all from Metabion International Ag): ladder-targeting (5′-IR800-AAGCAGGTCCTCGTCGCCGTACACCTCATCATACA-3′), SwSCV-1 genome-targeting (5′-IR800-GCTCTCCCCGGCAAGTCTTCTATTTCTGTCCTTCC-3′), SwSCV-1 antigenome-targeting (5′- IR800-GGAAGGACAGAAATAGAAGACTTGCCGGGGAGAGC-3′), or DAg mRNA-targeting (5′-IR800-TAATCTCTTTCGGTGGTAGCCTCGAGCCGCCATCC-3′) diluted in prehybridization buffer served for detection. We performed the hybridization O/N at 68 °C (using the genome-targeting probe) or 48 °C (using the antigenome- and mRNA-targeting probes), and washed the membrane once with 2× SSC and 0.1% SDS for 10 min at 68 °C/48 °C, followed by two 10 min washes with 1× SSC and 0.1% SDS at 68 °C/48 °C. After the washes, the Odyssey Infrared Imaging System (LI-COR Biosciences, Lincoln, NE, USA) served to record the results. The protocol was adapted from Miller et al., 2018 [44]. 

We generated a short (~850 nucleotides long) control RNA fragment using the SwSCV-1 FWD plasmid described in Szirovicza et al., 2020 [17]. We used FastDigest EcoRV and Acc65I (ThermoFisher Scientific, Waltham, MA, USA) to linearize and digest the plasmid. Then, we purified the fragment of interest—containing the T7 promoter—from agarose gel using the GeneJet Gel Purification Kit. The plasmid fragment was further purified using SPRIselect magnetic beads (Beckman Coulter, Brea, CA, USA). TranscriptAid T7 High Yield Transcription Kit (ThermoFisher Scientific, Waltham, MA, USA) then served for in vitro transcription of the target RNA according to the manufacturer’s protocol. Finally, we cleaned up the RNA using the GeneJET RNA Purification Kit (ThermoFisher Scientific, Waltham, MA, USA) and stored the RNA at −80 °C until further usage. The control RNA fragment contains the target sequence for the SwSCV-1 DAg mRNA probe described above.

### 2.9. SwSCV-1 Infection Dynamics in Naïve I/1Ki Cells

We superinfected 2× and 1.2× SwSCV-1 FWD transfected I/1Ki cells (~6 months post transfection) with HISV-1 (MOI of 10, as described earlier), and collected the supernatants from both superinfected cell lines at 3 dpi. We used 1:5 and 1:100 diluted supernatants to inoculate naïve I/1Ki cells plated on 24-well plates (for WB and qRT-PCR) and 96-well plates (for IF staining), and collected samples for WB, qRT-PCR, and IF staining at 3, 6, and 9 dpi, as described above. 

## 3. Results

### 3.1. Transfection with 1.2× Genome Construct Initiates Replication of Kolmiovirids

In our previous study, we showed in a transfection-based assay that a plasmid bearing the SwSCV-1 genome in duplicate could initiate SwSCV-1 replication in cell culture, with highest efficacy observed in boid kidney cells [17]. We hypothesized, based on RNA transfection studies with HDV [11], that a plasmid bearing a shorter 1.2× genome-length insert would suffice to initiate translation. With the idea that duplicating the antigenomic and genomic ribozymes would better facilitate replication, we ordered synthetic genes representing the 1.2× genome of SwSCV-1, TSRV-1, DabDV-1, CITV-1, and HDV-1. Figure 1 shows the organization of the synthetic blocks that we subsequently cloned in both reverse and forward orientation into a pCAGGS/MCS expression vector under the CAG promoter. Unlike in our earlier study, in which we included an additional T7 promoter in the antigenomic orientation, upstream of the DAg to the synthetic construct [17], we did not include additional promoters that could unintentionally facilitate DAg translation. The resulting constructs we named according to the 1.2× “kolmiovirid” FWD and 1.2× “kolmiovirid” REV scheme. The FWD constructs drive transcription of the respective kolmiovirid genome, due to which the expression or translation of the DAg should only occur following virus replication, since the DAg ORF is in antigenomic orientation. On the other hand, the REV constructs would generate antigenomic transcripts that would likely also mediate the DAg translation.

**Figure 2:**
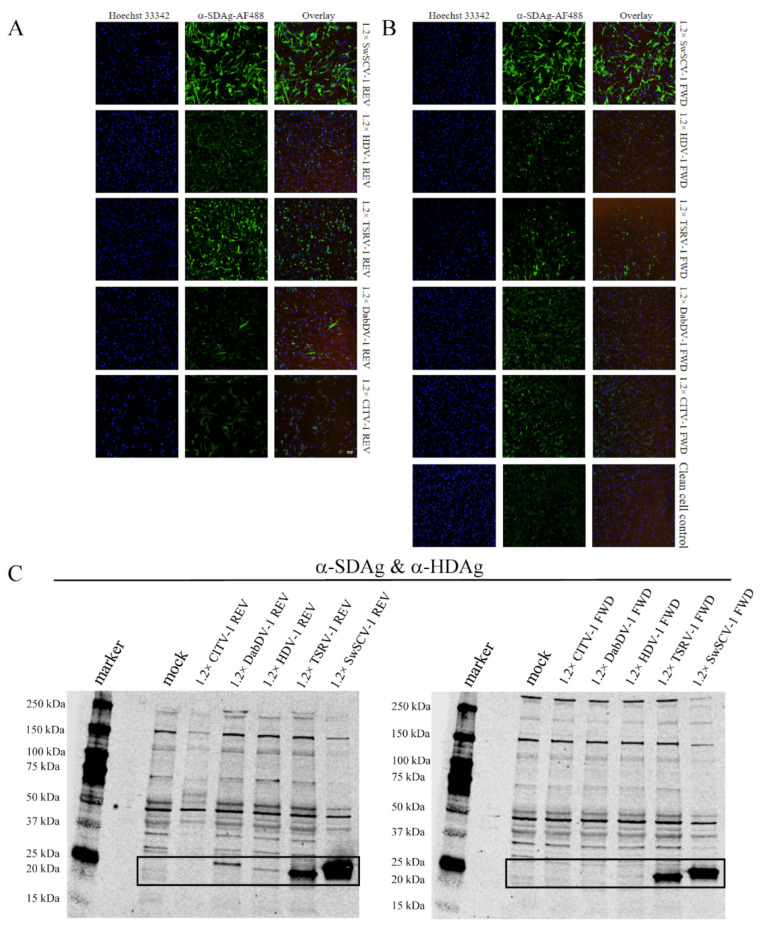
SwSCV-1 DAg antiserum cross-reactivity with the DAg of different kolmiovirids. (**A**) I/1Ki cells transfected with 1.2× SwSCV-1, HDV-1, TSRV-1, DabDV-1, and CITV-1 REV constructs were stained for the DAg at 4 days post transfection using rabbit α-SwSCV-1 DAg antiserum (1:100 dilution). (**B**) I/1Ki cells transfected with 1.2× SwSCV-1, HDV-1, TSRV-1, DabDV-1, and CITV-1 FWD constructs and clean cell control were stained for the DAg 4 days post transfection using rabbit α-SwSCV-1 DAg antiserum (1:100 dilution). Hoechst 33342 served for detection of the nuclei (**left panels**), and AlexaFluor 488-labeled donkey anti-rabbit IgG as the secondary antibody for DAg detection (**middle panels**). The (**right panels**) show overlay of the nuclear and DAg staining. The images were captured using Opera Phenix High Content Screening System (PerkinElmer, Waltham, MA, USA) with 20× objective. (**C**) I/1Ki cells transfected with 1.2× SwSCV-1, HDV-1, TSRV-1, DabDV-1, and CITV-1 REV constructs (**left panel**) and FWD constructs (**right panel**) were submitted for western blot at 4 days post transfection. The samples were separated on 4–20% Mini-PROTEAN TGX gels (Bio-Rad, Hercules, CA, USA), transferred onto nitrocellulose, and the membranes were probed with rabbit α-SwSCV-1 DAg antiserum and affinity purified α-HDAg antibody. We loaded 1/3 volume of the 1.2× SwSCV-1 REV and FWD samples. The bands corresponding to the different DAgs are marked with the black rectangle. The results were recorded using Odyssey Infrared Imaging System (LI-COR Biosciences, Lincoln, NE, USA).

To test if the shorter constructs will facilitate replication, and to estimate the cross-reactivity of our rabbit anti-SwSCV-1 DAg antiserum, we transfected I/1Ki cells with each of the constructs. IF staining of the cells transfected with REV constructs for DAg at 4 days post transfection (dpt) showed that the anti-SwSCV-1 DAg antiserum clearly cross-reacted with TSRV-1 and HDV-1, but also to some extent with DabDV-1 and CITV-1 (Figure 2A). We detected DAg in cells transfected with the FWD constructs of HDV-1, TSRV-1, and SwSCV-1, suggesting that transfection resulted in replication initiation (Figure 2B). The staining for DAg of DabDV and CITV-1 was much less prominent on the cells transfected with FWD construct; however, the results suggest that replication occurs also with these viruses. We also performed western blot (WB) on the transfected cells and we were able to detect DAgs of SwSCV-1, HDV-1, TSRV-1, and DabDV-1 4 days after transfection with the REV constructs. However, after transfection with the FWD constructs, we were only able to detect bands for DAgs of SwSCV-1, HDV-1, and TSRV-1 (Figure 2C). The observed differences between the results of IF staining and WB could be due to the ability of the antibody to bind conformational epitopes in case of IF staining; however, in WB this would be less likely.

### 3.2. The 1.2× and 2× SwSCV-1 Genome Infectious Clones Induce Similar Infection as Judged by Antigen Expression and Replication 

**Figure 3:**
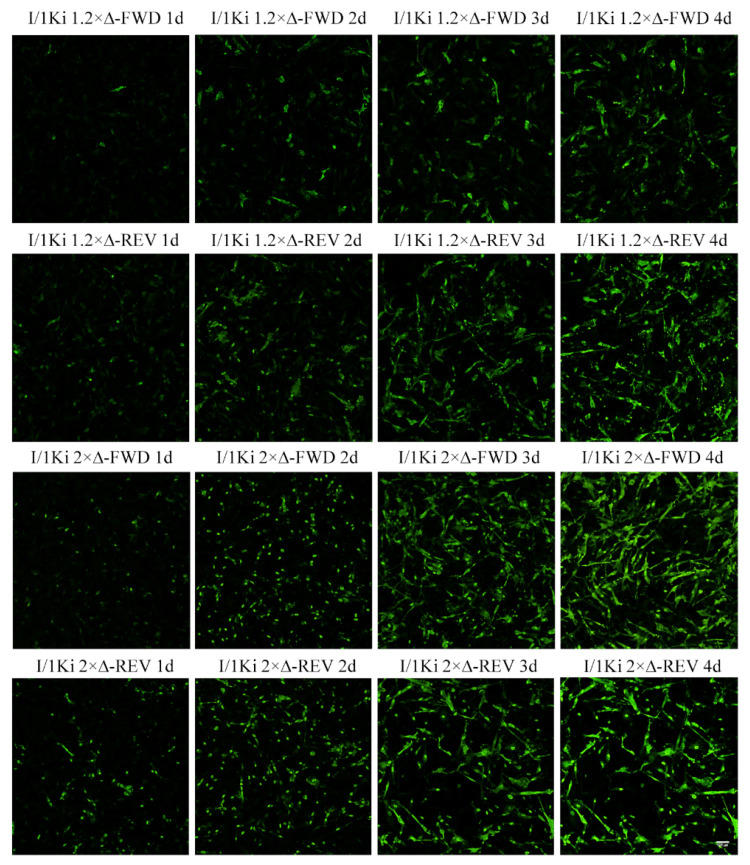
Expression of DAg in I/1Ki cells following transfection with 2× and 1.2× genome SwSCV-1 FWD and REV plasmids. I/1Ki cells transfected with 2× and 1.2× SwSCV-1 FWD and REV plasmids were fixed and stained for the DAg using rabbit α-SwSCV-1 DAg antiserum at 1–4 days post transfection. AlexaFluor 488-labeled donkey anti-rabbit IgG served as the secondary antibody for DAg detection. The images were captured using Opera Phenix High Content Screening System (PerkinElmer, Waltham, MA, USA) with 20× objective.

To compare the ability of the shorter 1.2× genome length constructs to initiate replication, we transfected I/1Ki cells with 1.2× SwSCV-1 FWD and 1.2× SwSCV-1 REV and compared the antigen expression and SwSCV-1 RNA production between 1 and 5 dpt. IF staining of the transfected cells at 1–4 dpt demonstrates that both 1.2× and 2× genome constructs efficiently drive the expression of DAg (Figure 3). 

**Figure 4:**
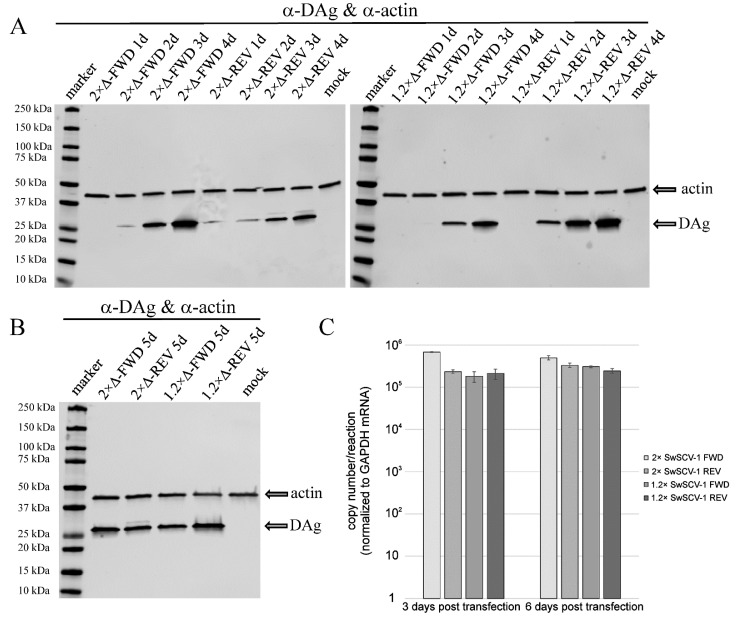
Western blot of I/1Ki cells after transfection with 2× and 1.2× SwSCV-1 (2×Δ and 1.2×Δ, respectively) FWD and REV constructs. (**A**) Samples of I/1Ki cells transfected with 2×Δ-FWD, 2×Δ-REV, 1.2×Δ-FWD, and 1.2×Δ-REV constructs collected at 1–4 days post transfection were separated on 4–20% Mini-PROTEAN TGX gels (Bio-Rad, Hercules, CA, USA), transferred onto nitrocellulose, and the membranes were probed with rabbit α-SwSCV-1 DAg antiserum and mouse monoclonal anti-pan actin antibody. The left panel shows 2× and the right panel 1.2× genome constructs. The results were recorded using Odyssey Infrared Imaging System (LI-COR Biosciences, Lincoln, NE, USA). (**B**) Samples of I/1Ki cells transfected with 2×Δ-FWD, 2×Δ-REV, 1.2×Δ-FWD, and 1.2×Δ-REV constructs collected at 5 days, analyzed as described in (**A**). (**C**) RNA isolated from I/1Ki cells transfected with 2×Δ-FWD, 2×Δ-REV, 1.2×Δ-FWD, and 1.2×Δ-REV constructs at 3 and 6 days post transfection were analyzed by qRT-PCR targeting genomic SwSCV-1 RNA. In vitro transcribed RNA target served for obtaining a standard curve to convert cycle threshold values into copy numbers. qRT-PCR targeting GAPDH mRNA served for normalizing the results between samples. The y-axis shows copy numbers/reaction. The error bars represent standard deviation.

To better estimate the amount of DAg produced by the different constructs, we performed WB on samples collected between 1 and 4 dpt. The results showed that similarly to the 2× genome constructs, the transfection of 1.2× constructs led to an increasing amount of DAg expression during the interval studied (Figure 4A). As the amount of DAg appeared to increase until 4 dpt and because the results of the IF staining suggested minute differences in the amount of DAg expression through the different construct, we performed WB analysis of samples collected at 5 dpt. The results indicate that the DAg expression level in the cells is similar at 5 dpt, regardless of the construct used for transfection (Figure 4B). DAg expression in REV constructs could be driven by the CAG promoter of the plasmid, but since levels were similar at 5 dpt, we interpret the result to suggest that DAg expression is due to replication. To add another dimension to the determination of the replication efficiency, we analyzed I/1Ki cells transfected with the four different constructs for SwSCV-1 RNA levels at 3 and 6 dpt by qRT-PCR. The 2× SwSCV-1 (especially the FWD) genome construct appeared to initiate replication more rapidly as demonstrated by the higher amount of SwSCV-1 RNA at 3 dpt (Figure 4C). However, at 6 dpt, the cells transfected with each of the constructs showed similar SwSCV-1 RNA levels when normalized against GAPDH mRNA (Figure 4C), supporting the WB-based interpretation that all of the studied constructs can initiate replication.

### 3.3. Superinfection of Cells Transfected with 1.2× SwSCV-1 FWD Construct Induces Infectious Particle Formation

**Figure 5:**
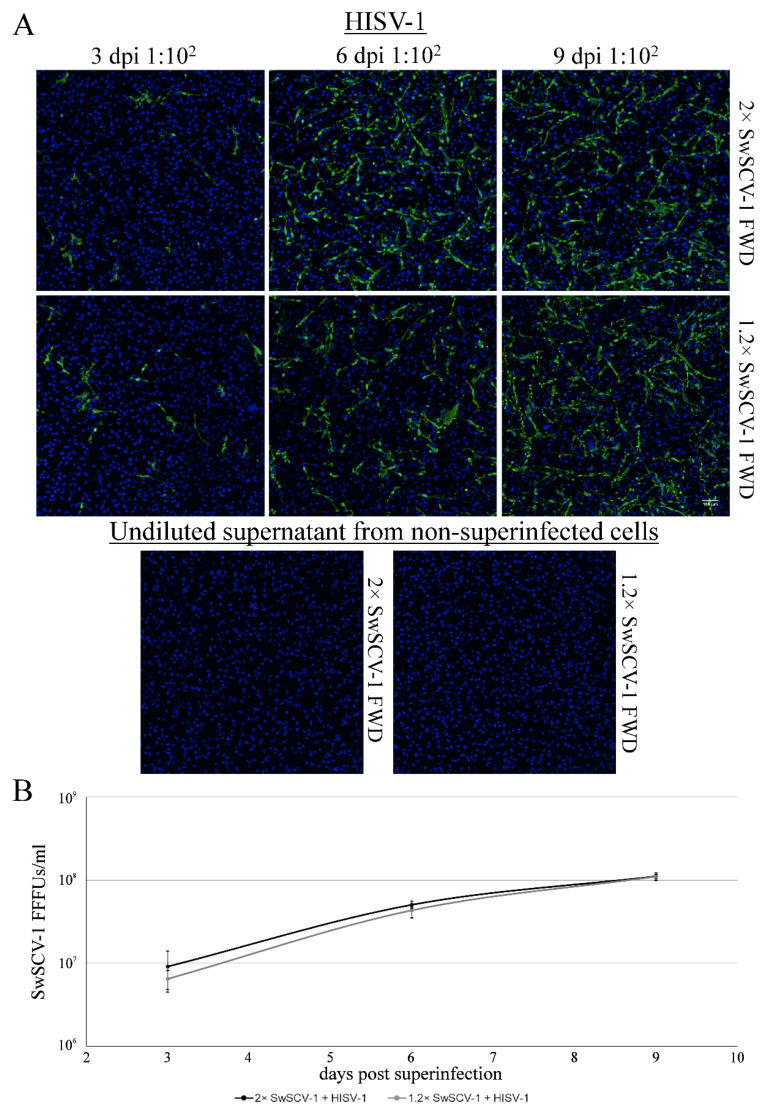
Superinfection of 2× and 1.2× SwSCV-1 FWD transfected I/1Ki cells leads to infectious particle production. (**A**) Supernatants collected at 3, 6, and 9 days post HISV-1 superinfection from I/1Ki cells—transfected with 2× and 1.2× SwSCV-1 FWD constructs two weeks earlier—were used to inoculate clean I/1Ki cells. At four days post inoculation, the cells were fixed and stained using rabbit α-SwSCV-1 DAg antiserum and Alexa Fluor 488-labeled donkey anti-rabbit secondary antibody. Hoechst 33342 served for staining the nuclei. The top panels show clean I/1Ki cells infected with 100-fold diluted supernatant originating from HISV-1 superinfected 2× SwSCV-1 FWD transfected cells, and the bottom panels with supernatant originating from HISV-1 superinfected 1.2× SwSCV-1 FWD transfected cells. Undiluted supernatant from non-superinfected cells served as a control. The images were captured using Opera Phenix High Content Screening System (PerkinElmer, Waltham, MA, USA) with 20× objective. (**B**) Opera Phenix High Content Screening System (PerkinElmer, Waltham, MA, USA) served to count the number of infected cells in (**A**), which enabled the quantification of infectious particles per milliliter of growth medium in terms of fluorescent focus-forming units (FFFUs—displayed on y-axis). The error bars represent standard deviation.

To show that the 1.2× SwSCV-1 construct not only initiates virus replication in cell culture, but also induces infectious particle formation in the presence of a suitable helper virus, we superinfected 1.2× and 2× SwSCV-1 FWD transfected cells with HISV-1, a hartmanivirus demonstrated to act as a helper for SwSCV-1 [17]. We titrated the supernatants collected at 3, 6, and 9 dpi with HISV-1 on clean I/1Ki cells and used supernatants collected from non-superinfected cells as the control. IF staining of cells inoculated with the supernatants at 4 dpi for the DAg served for detecting the infected cells (Figure 5A). We determined the number of infectious units by counting the fluorescent foci at each time point, and the results showed I/1Ki cells transfected with 1.2× or 2× SwSCV-1 FWD constructs to be equally effective in producing infectious particles following superinfection (Figure 5B). As observed for 2× SwSCV-1 FWD in our earlier study [17], the non-superinfected 1.2× SwSCV-1 FWD cells were not able to produce infectious SwSCV-1 particles (Figure 5A).

### 3.4. Transfection of Cells with the 1.2× SwSCV-1 Construct Results in Persistent Infection

**Figure 6:**
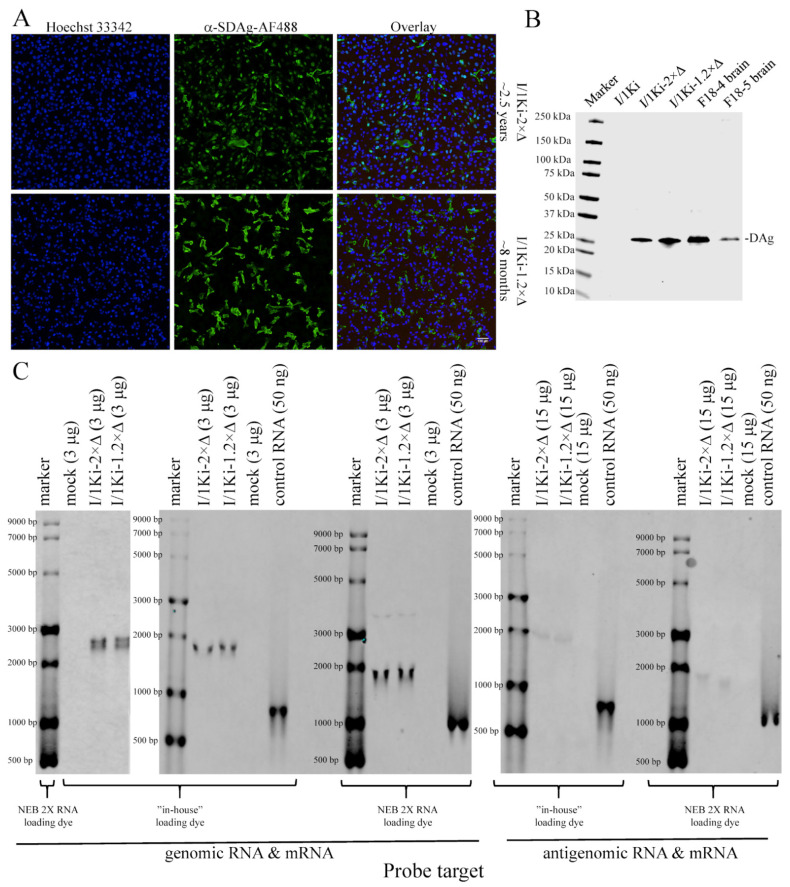
Comparison of persistently SwSCV-1-infected I/1Ki cells generated following transfection with 2× and 1.2× SwSCV-1 FWD constructs by immunofluorescence, and western and northern blot. The 2× SwSCV-1 (I/1Ki-2×Δ) cell line was analyzed at approximately 2.5 years and the 1.2× SwSCV-1 (I/1Ki-1.2×Δ) at approximately 8 months after initial transfection, during which the cell lines were passaged at 1–2 week interval. (**A**) Rabbit α-SwSCV-1 DAg antiserum and Alexa Fluor 488-labeled donkey anti-rabbit secondary antibody served for IF staining of the fixed cells, and Hoechst 33342 for staining the nuclei. The top panels show staining of I/1Ki-2×Δ cells, and the bottom panels the staining of I/1Ki-1.2×Δ cells. The left panels show staining of nuclei in blue, the middle panels show DAg staining in green, and the right panels show an overlay. The images were captured using Opera Phenix High Content Screening System (PerkinElmer, Waltham, MA, USA) with 20× objective. (**B**) Samples of naïve I/1Ki cells, I/1Ki-2×Δ cells, I/1Ki-1.2×Δ cells, and the brain homogenates of SwSCV-1-infected boa constrictors (F18-4 and F-18-5, of [15]) were separated on 4–20% Mini-PROTEAN TGX gels (Bio-Rad, Hercules, CA, USA), transferred onto nitrocellulose, and the membranes probed with rabbit α-SwSCV-1 DAg antiserum and mouse monoclonal anti-pan actin antibody. The results were recorded using Odyssey Infrared Imaging System (LI-COR Biosciences, Lincoln, NE, USA). (**C**) Indicated amounts of total RNA isolated from I/1Ki-2×Δ, I/1Ki-1.2×Δ, and clean I/1Ki cells and an in vitro-transcribed control RNA (~850 nucleotides long) were prepared using two different loading dyes (2X RNA loading dye [NEB] or “in-house” loading dye prepared according to Mansour and Pestov [43]), separated on agarose gel and transferred onto nylon membrane. Probes were targeting SwSCV-1 genomic RNA and SwSCV-1 DAg mRNA (left and middle panels) and antigenomic RNA and SwSCV-1 DAg mRNA (right panel); the bands of the marker served for visualizing the RNA targets. The results were recorded using Odyssey Infrared Imaging System (LI-COR Biosciences, Lincoln, NE, USA).

In our previous study, we showed that by maintaining I/1Ki cells after transfection with the 2× SwSCV-1 FWD construct, we could generate persistently SwSCV-1-infected cell lines [17]. At the time of preparing this manuscript, we have maintained the I/1Ki-2×Δ cell line for 2.5 years, and IF staining for DAg shows the cell line to be persistently SwSCV-1-infected (Figure 6A). To compare the replication behavior of the shorter construct further, we transfected I/1Ki cells with 1.2× SwSCV-1 FWD and continued passaging the cells. Analysis of the cells by IF staining for DAg at 8 months post initial transfection indicates that also the 1.2× SwSCV-1 FWD construct can induce persistent infection in I/1Ki cells (Figure 6A). We compared the generated cell line, I/1Ki-1.2×Δ, to I/1Ki-2×Δ cells further by analyzing the amount of DAg expression using WB. The results show that DAg expression by I/1Ki-1.2×Δ cells is at least at the level observed in I/1Ki-2×Δ cells (Figure 6B), supporting the observation of a similar replication efficiency. To further compare the cell lines, we set up a near-infrared fluorescent northern blot assay for detection of the genomic RNA, antigenomic RNA, and DAg mRNA. As an additional control, we included an in vitro transcribed RNA of approximately 850 nucleotides corresponding roughly to the size of SwSCV-1 DAg mRNA. Initially, we prepared the samples for the denaturing agarose gel run by using an “in-house” loading dye described by Mansour and Pestov [43], but ran the RNA marker with the loading dye provided by New England Biolabs (NEB). To our surprise, the northern blot of RNA isolated from I/1Ki-1.2×Δ and I/1Ki-2×Δ cells using a probe targeting the genomic RNA resulted in the detection of a doublet band migrating at around 2.8 kilonucleotides (knt) instead of the expected 1.7 knt as compared to the RNA marker (Figure 6C left panel). To study if the use of two different loading dyes had significantly affected the migration of the RNA, we ran the RNAs extracted from I/1Ki-2×Δ, I/1Ki-1.2×Δ, and clean cells as well as the in vitro-transcribed control RNA and the RNA marker in parallel with both loading dyes. Indeed, the result showed the loading dye to significantly affect the migration of the RNA, and indicated that the SwSCV-1 genomic RNA is approximately 1.7 knt in size as judged by migration (Figure 6C). Therefore, we speculate that the doublet bands observed in the initial run likely correspond to the circular and nicked forms of the genomic RNA. With the probe targeting genomic RNA, we also detected a band migrating at around 3.4 knt, which likely represents the genome dimer reported to be present in the infected cells by other researchers [45]. In order to detect antigenomic RNA from the persistently infected cells, we had to load 5 times more RNA, which corresponds roughly to the ratio of genomic and antigenomic RNA reported for HDV [46]. We were unable to detect DAg mRNA in the persistently infected cells, even though the probe detected the in vitro transcribed control RNA (Figure 6C). The result thus suggests that the amount of DAg mRNA in the persistently infected cells is below our detection limit.

**Figure 7:**
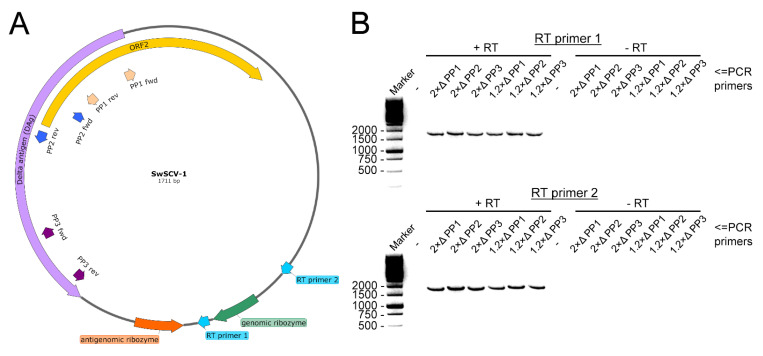
Demonstration of circular SwSCV-1 genome in persistently infected cells using two-step RT-PCR. (**A**) We transcribed cDNA with primers targeting the genomic RNA upstream of either both genomic and antigenomic ribozyme (RT primer 1) or just the antigenomic ribozyme (RT primer 2) to include the putative cleavage sites of the genomic RNA. The subsequent PCR employed three different primer pairs (PP1–PP3) targeting the DAg ORF to amplify the nearly complete SwSCV-1 genome. The figure shows the location of primers in the SwSCV-1 genome map. (**B**) The PCR products with PP1 to PP3 from templates produced from the RNAs extracted from I/1Ki-2×Δ and I/1Ki-1.2×Δ in the presence (left half of both gels) or absence (right side of both gels) of RT enzyme. The top panel shows PCR products with RT primer 1 and the bottom with RT primer 2 separated on 1.2% agarose gel with GelRed for visualization of the bands, the expected size of the amplicons is roughly 1650 nt.

The HDV RNA genome is circular [6], and we wanted to study if the SwSCV-1 RNA genome in the persistently infected cells shares this characteristic feature. To that end, we designed RT primers to transcribe cDNA going over the potential cleavage sites of the genomic RNA using RNA extracted from I/1Ki-2×Δ and I/1Ki-1.2×Δ cells as the template (Figure 7A—RT primer 1 and 2). To show that the genome is circular, we designed three primer pairs (PPs) targeting a region that is continuous with certainty, i.e., the DAg ORF. The PPs designed have their 3′ ends facing opposite directions on the reverse-complementary template strands (Figure 7A—PP1 to PP3). As a control, we performed the exact same reactions with the same set of templates and primers, but without the addition of the RT enzyme. With both RT primers and PPs 1–3, we succeeded in amplifying the near complete SwSCV-1 genome, from templates generated in the presence of RT enzyme, indicating that the SwSCV-1 genome is indeed circular (Figure 7B). 

### 3.5. Inoculation of Naïve I/1Ki Cells with SwSCV-1 Results in Productive

Lastly, we wanted to study if SwSCV-1 released from cells originally transfected with 1.2× and 2× SwSCV-1 FWD (experiment conducted 6 months post transfection) superinfected with HISV-1 would result in productive infection in naïve I/1Ki cells. We used supernatants collected at 3 dpi from the HISV-1 superinfected cells (earlier observed to contain adequate amount of infectious SwSCV-1, Figure 6A) at two dilutions, 1:5 and 1:100, to inoculate naïve I/1Ki cells, and collected samples from the inoculated cells at 3, 6, and 9 dpi. IF staining, qRT-PCR, and WB served to monitor, respectively, the increase in the number of infected cells, SwSCV-1 RNA, and DAg within the cells. 

**Figure 8:**
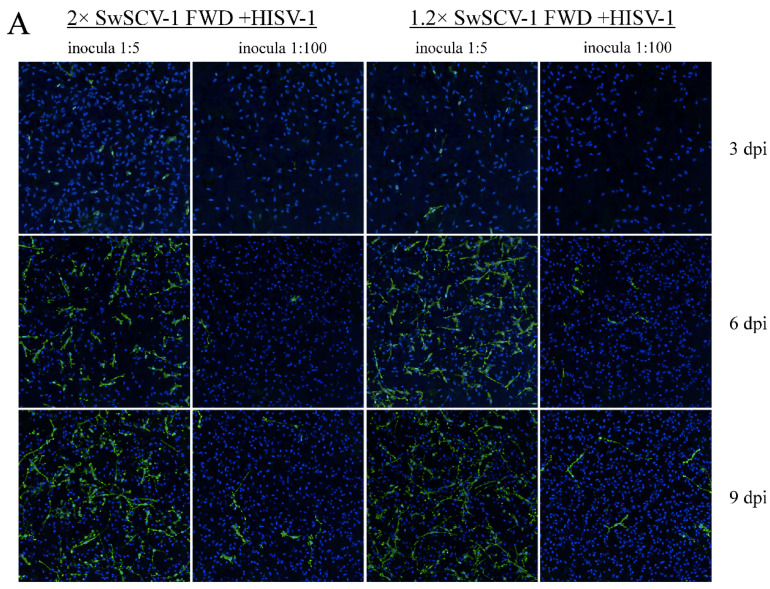

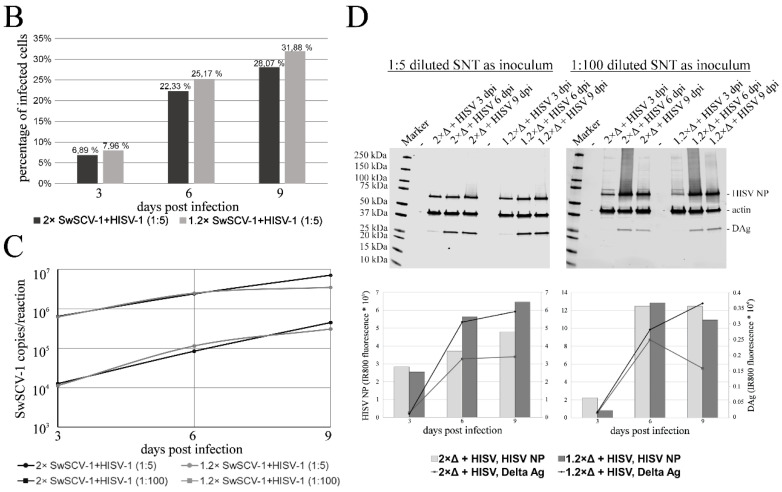
SwSCV-1 infection on naïve I/1Ki cells. Supernatants from I/1Ki cells transfected six months ago with 1.2× or 2× SwSCV-1 FWD were collected three days post superinfection with HISV-1 and subsequently used to inoculate naïve I/1Ki cells at 1:5 and 1:100 dilutions. (**A**) Rabbit α-SwSCV-1 DAg antiserum and Alexa Fluor 488-labeled donkey anti-rabbit secondary antibody served for IF staining of the fixed cells, and Hoechst 33,342 for staining the nuclei. The left panels show an overlay of DAg (green) and nuclear (blue) staining of I/1Ki-2×Δ cells and the right panels the staining of I/1Ki-1.2×Δ cells fixed at 3, 6, or 9 dpi. The images were captured using Opera Phenix High Content Screening System (PerkinElmer, Waltham, MA, USA) with 20× objective. (**B**) Opera Phenix High Content Screening System (PerkinElmer, Waltham, MA, USA) served for enumerating the number of infected cells at each time point. The dark bars represent cells inoculated with 1:5 dilution of HISV-1 superinfected 2× SwSCV-1 and the light bars cells inoculated with 1:5 dilution of HISV-1 superinfected 1.2× SwSCV-1 cell culture supernatant. (**C**) RT-PCR served to quantify the amount of SwSCV-1 RNA in the cells at each time point. The number of SwSCV-1 RNA copies in the reaction (corresponding to 1/20 of RNA extracted from cells of a single 24-well plate well) normalized against housekeeping gene (GAPDH). (**D**) Samples of cells inoculated with 1:5 or 1:100 diluted supernatant collected from HISV-1 superinfected 2× SwSCV-1 FWD or 1.2× SwSCV-1 FWD transfected cells were separated on 4–20% Mini-PROTEAN TGX gels (Bio-Rad, Hercules, CA, USA), transferred onto nitrocellulose, and the membranes probed with rabbit α-SwSCV-1 DAg antiserum, rabbit α-HISV NP antiserum, and mouse monoclonal anti-pan actin antibody. The top panels show the results recorded using Odyssey Infrared Imaging System (LI-COR Biosciences, Lincoln, NE, USA), and the bottom panels show results for the quantification (using Image Studio Lite Ver 2) of the HISV NP and DAg bands normalized against the actin signal.

IF staining of the inoculated cells for DAg at 3 dpi showed prominent nuclear staining; however, at 6 and 9 dpi, after the infection had properly established and spread to new cells, DAg showed more pronounced cytoplasmic staining (Figure 8A). To assess the spread of infection, we quantified the number of infected cells at each time point based on the IF staining for DAg. The increase in the number of infected cells and the DAg amount coincided with the increase in the amount of SwSCV-1 RNA in the cells as studied by qRT-PCR (Figure 8C). The number of infected cells increased roughly 4-fold during the course of the infection (Figure 8B). WB from the cell pellets collected 3, 6, and 9 dpi showed an increase in the amount of DAg over the course of the experiment (Figure 8D).

## 4. Discussion

The identification of novel HDV-like agents, significantly divergent from HDV [14,15,18,19,20,21], which until 2018 was the sole representative of the previously unassigned genus *Deltavirus*, has increased the interest in HDV and kolmiovirid research. The identification of HDV-like agents in various host species without traces of hepadnaviruses by others and us led to questioning the strict association of HDV and HBV. Co-incidentally, Perez-Vargas and colleagues showed that HDV is able to use helper viruses other than HBV to form infectious particles [16]. We demonstrated that SwSCV-1 efficiently utilized reptarena- and hartmaniviruses as its helpers, and that the co-expression of different arena- and orthohantavirus glycoproteins can drive infectious particle formation [17]. Construction of infectious clones is the first step in demonstrating that the sequences recovered through metatranscriptomic analyses are indeed complete and capable of driving replication. We reported generation of such a clone by inserting two copies of the SwSCV-1 genome in head-to-tail fashion into a mammalian expression vector, pCAGGS [17]. The same approach was proven functional for TSRV-1 [19] as well as for the HDV-like agents in *Taeniopygia guttata* and *Marmota monax* [21]. The first HDV infectious clone contained a trimeric HDV genome, and the authors utilized a dimeric genome-containing plasmid with deletion in the DAg ORF to demonstrate the protein’s role in replication [4]. Constructs containing multiples of the genome make synthetic inserts longer and complicate mutational studies because each modification needs to be inserted/generated multiple times. This motivated us to attempt generation of 1.2× genome infectious clones for initiation of kolmiovirid replication. The availability of tools and reagents at hand forced us to focus on comparing the replication initiation between 2× and 1.2× SwSCV-1 genome clones in depth, but we were also able to demonstrate that a similar approach might work for the recently identified kolmiovirids.

The replication of HDV occurs via rolling circle replication by cellular RNA polymerases [11], during which the genomic and antigenomic ribozymes cut the produced genome multimers into unit-length pieces [47]. The recently identified kolmiovirids presumably share the same replication strategy and possess the genomic and antigenomic ribozymes [14,15,19,21,48]. Based on the HDV literature [49,50,51], we reasoned that duplicating the genomic and antigenomic ribozyme sequences would facilitate initiation of replication and/or production of unit-length genome (and antigenome). Indeed, RNA transfection studies with HDV have shown 1.2× genome copies to be most efficient in induction of virus replication [29], and a similar approach has been applied to generate HDV infectious clones, although we were unable to decipher the exact organization of the constructs [30,31,52]. By applying the same principle, we constructed 1.2× genome infectious clones for HDV-1, SwSCV-1, TSRV-1, DabDV-1, and CITV-1 in both genomic and antigenomic sense, and tested the clones in I/1Ki cells, which efficiently support replication of SwSCV-1 following transfection with the 2× genome clone [17]. In the REV constructs, the CAG promoter of the pCAGGS vector should mediate DAg translation, which provides a source of DAg for the first rounds of replication. For HDV, the reports suggest existence of an internal promoter that could drive the production of DAg [28,53], such a promoter would presumably act in both our REV and FWD constructs and could also contribute to replication initiation. Indeed, the comparison of DAg production following transfection with 1.2× and 2× genome SwSCV-1 FWD and REV constructs demonstrated detectable DAg levels to appear earlier in cells transfected with REV constructs (Figure 4A). We thus used the IF staining of DAg from I/1Ki cells transfected with the 1.2× genome REV constructs (HDV-1, TSRV-1, DabDV-1, and CITV-1) to estimate the cross-reactivity of the anti-SwSCV-1 DAg antiserum [15] with DAg of the different viruses. The antiserum appeared to cross-react best with TSRV-1 DAg that is the closest relative of SwSCV-1 from the kolmiovirids included [19]. The fact that HDV-1 DAg showed prominent nuclear staining, as would be expected based on the HDV literature [54], increased our confidence in the specificity of the IF staining. The antiserum appeared to cross-react moderately well with the DAg of DabDV-1, showing mainly cytoplasmic staining. While the CITV-1 DAg appeared to be barely detectable with the antiserum, the results did suggest production of CITV-1 DAg. Although the expression of DAg could come from internal promoters, we think that the DAg produced following transfection of FWD constructs is due to initiation of replication. While the inability of our SwSCV-1 DAg antiserum to cross-react with the DAgs of the other viruses tested likely explains the lower signal, it is also likely that the different kolmiovirids are not replicating optimally in the *B. constrictor* cells. We hypothesize that each of the kolmiovirids would be through promoter usage adapted to replicating in a specific host species.

Along with our data on the ability of 1.2× and 2× genome SwSCV-1 constructs to initiate replication and to induce a persistent infection, we demonstrated the presence of circular SwSCV-1 RNA in the infected cells. We also demonstrated that inoculation of naïve I/1Ki cells with supernatants containing infectious SwSCV-1 and a suitable helper virus results in productive infection. These observations strongly support our conclusion that plasmid transfection of I/1Ki cells indeed efficiently initiates SwSCV-1 replication.

The 1.2× genome construct design described herein (analogous to constructs described for HDV) could help to reduce the complexity of introducing mutations, and facilitate synthetic gene design for molecular biology studies of the recently identified kolmiovirids and those that will be identified in the future. Our results with SwSCV-1 show that the 1.2× genome clone is at least as efficient as the 2× genome clone in initiation of replication. Furthermore, our results indicate that introduction of the insert in either genomic or antigenomic orientation functions equally well in the *B. constrictor* kidney cell model. Further studies with HDV-1, TSRV-1, DabDV-1, and CITV-1 in cell lines of various species could serve to demonstrate species specificity of the viruses, and to provide first evidence on the potential role of kolmiovirid promoters in mediating species-specific replication.

## Data Availability

Not applicable.
